# Medical Emergency Team syndromes and an approach to their management

**DOI:** 10.1186/cc4821

**Published:** 2006-02-15

**Authors:** Daryl Jones, Graeme Duke, John Green, Juris Briedis, Rinaldo Bellomo, Andrew Casamento, Andrea Kattula, Margaret Way

**Affiliations:** 1 Intensive Care Unit, The Alfred Hospital, Commercial Road Melbourne, Victoria, Australia, 3004; 2 Intensive Care Unit, The Northern Hospital, Cooper Street Epping, Victoria, Australia, 3076; 3 Department of Anaesthesia, The Northern Hospital, Cooper Street Epping, Victoria, Australia, 3076; 4 Department of Intensive Care and Department of Surgery, The Austin Hospital, Studley Road Heidelberg, Victoria, Australia, 3084; 5 Department of Strategy Risk and Clinical Governance, The Austin Hospital, Studley Road Heidelberg, Victoria, Australia, 3084

## Abstract

**Introduction:**

Most literature on the medical emergency team (MET) relates to its effects on patient outcome. Less information exists on the most common causes of MET calls or on possible approaches to their management.

**Methods:**

We reviewed the calling criteria and clinical causes of 400 MET calls in a teaching hospital. We propose a set of minimum standards for managing a MET review and developed an approach for managing common problems encountered during MET calls.

**Results:**

The underlying reasons for initiating MET calls were hypoxia (41%), hypotension (28%), altered conscious state (23%), tachycardia (19%), increased respiratory rate (14%) and oliguria (8%). Infection, pulmonary oedema, and arrhythmias featured as prominent causes of all triggers for MET calls. The proposed minimum requirements for managing a MET review included determining the cause of the deterioration, documenting the events surrounding the MET, establishing a medical plan and ongoing medical follow-up, and discussing the case with the intensivist if certain criteria were fulfilled. A systematic approach to managing episodes of MET review was developed based on the acronym '**A to G**': **a**sk and **a**ssess; **b**egin **b**asic investigations and resuscitation, **c**all for help if needed, **d**iscuss, **d**ecide, and **d**ocument, **e**xplain aetiology and management, **f**ollow-up, and **g**raciously thank staff. This approach was then adapted to provide a management plan for episodes of tachycardia, hypotension, hypoxia and dyspnoea, reduced urinary output, and altered conscious state.

**Conclusion:**

A suggested approach permits audit and standardization of the management of MET calls and provides an educational framework for the management of acutely unwell ward patients. Further evaluation and validation of the approach are required.

## Introduction

Medical emergency team (MET) systems have been introduced into hospitals to identify, review and treat acutely unwell ward patients. Most of the literature related to METs has concentrated on their effects in reducing cardiac arrests and serious adverse events [1], primarily in single-centre studies. However, a recent Australian multi-centre cluster-randomized trial failed to confirm that the introduction of METs into hospitals was able to improve these outcomes [2]. Despite this negative result, substantial interest in the utility of METs has developed in both the USA and the UK.

Limited information exists on the causes and outcomes of episodes of MET reviews. There is even less information on the process of assessment and management undertaken by the MET during an episode of MET review. To our knowledge, no information exists on a systematic approach to managing MET calls.

It is likely that a limited number of conditions precipitate MET calls [3] and that a MET syndrome or several MET syndromes exist [1].

We present here a systematic approach for the assessment and management of problems commonly encountered during an episode of MET review.

## Methods

### The hospitals

The Northern and Austin Hospitals are both situated in the north of Melbourne and are affiliated with the University of Melbourne. The Northern Hospital provides acute and elective medical services, except cardiac surgery, neurosurgery and organ transplantation. The Austin Hospital provides all acute and elective medical services and is the referral centre for liver transplantation and spinal cord injuries for the state of Victoria. The Northern Hospital has a 10-bed intensive care unit (ICU) that is staffed by an intensive care registrar during the day, and a senior hospital medical officer and anaesthetic registrar overnight. The Austin Hospital has a 21-bed ICU that is staffed by intensive care registrars at all times. In both hospitals the ICU medical staff may have a background in anaesthesia, internal medicine or emergency medicine.

### Ethics approval

Approval for the introduction of the MET and for the collection of data related to it was obtained from the Hospital Research and Ethics Committee of both hospitals.

### Medical response teams

Both hospitals have two levels of emergency response. The traditional 'Code Blue' call is intended for the resuscitation of cardiac arrests and other acute life-threatening emergencies. It consists of an anaesthetic registrar, a coronary care registrar and nurse, an ICU registrar and nurse, and the medical registrar of the receiving unit of the day. The MET is intended to review all medical emergencies other than cardiac arrests; it has been described in detail previously [4]. It can be activated by any member of hospital staff according to predetermined criteria that are based primarily on abnormalities of vital signs and clinical status (Table 1 in Additional file 1).

At the Austin hospital the MET consists of an ICU registrar and nurse, as well as the Medical registrar of the receiving unit of the day. Previously, activation of the MET at the Northern Hospital resulted in notification of only the patient's parent unit doctors. As part of an ongoing programme to improve the use of the MET at the Northern Hospital, activation of the MET now results in notification of the medical registrar and the intensive care registrar or hospital medical officer.

### Details of MET calls

A detailed log book is maintained by the switchboard operators at both hospitals that records all medical emergency calls. At the Austin Hospital, case report forms are also completed by the ICU registrar at the end of each call. These forms document the parent unit of the patient as well as the indications for the MET call. Since March 2002 the registrar has also recorded a provisional diagnosis of what medical condition is thought to have caused the MET call. Details of 400 calls that occurred between April and October 2004 were manually entered into an Excel spreadsheet to provide details on the trigger and the presumed aetiology of the call. Data are presented as percentages or absolute number of calls. No assumptions are made in cases in which data on presumed diagnosis were missing.

### Proposed minimum standards for managing a MET call

The proposed minimum standards were developed after a series of meetings and electronic communications between all the authors of this manuscript. The '**A to G**' approach to managing a MET call was subsequently developed to achieve these minimal standards. Finally, the '**A to G**' approach was adapted to provide a plan for the management of the five most common 'MET syndromes': tachycardia, hypotension, dyspnoea and hypoxia, altered conscious state, and oliguria.

## Results

### Characteristics of 400 MET calls

Of the 400 MET calls, 23 had only the 'staff worried' criterion. Of the remainder, 248 had one listed physiological MET criterion, 105 had two, 23 had three and one patient had four criteria. The average number of listed MET criteria for the 400 MET calls was 1.3 (531 criteria for 400 calls).

The proportions of MET criteria triggering a call were hypoxia (41%), hypotension (28%), altered conscious state (23%), tachycardia (19%), increased respiratory rate (14%) and oliguria (8%). Of the 531 calling criteria for the 400 MET calls, 61 had no documented provisional diagnosis. Several common causes for these triggers were identified (Table 2 in Additional file 1). Infections (especially pneumonia; 125/531 criteria), cardiogenic shock or pulmonary oedema (104/531 criteria) and arrhythmias (51/531 criteria) were thought to be responsible for 53% (280/531) of all triggers for MET calls (Table 2 in Additional file 1).

### Proposed minimum standards for managing a MET call

The proposed minimum requirements for managing an episode of a MET review included the following: first, determining the cause of the deterioration; second, documenting the events surrounding the MET; third, establishing a medical plan and ongoing medical follow-up; and fourth, discussing the case with the intensivist if predefined criteria were fulfilled (Table 3 in Additional file 1). Requirements specific to the Austin Hospital also included automatic medical referral for surgical patients who remained on the ward after a MET call for a medical reason, and compulsory review of the patient by an intensivist for a patient requiring two MET reviews in a seven-day period.

### Approach to the management of a MET call

An approach to the management of a MET call was developed with the acronym '**A to G**' (Table 4 in Additional file 1). The members of the MET are encouraged to **a**sk the nurses the reason for the MET call (that is, what calling criteria initiated the MET call) and **a**ssess the patient for the aetiology of the deterioration before **b**eginning **b**asic resuscitation. They are also encouraged to **c**all for help if needed. After initial resuscitation and assessment, the staff are instructed to **d**iscuss the case with appropriate medical staff, **d**ecide where the patient should be managed, and **d**ocument the events surrounding the MET. Issues surrounding the resuscitation status of the patient should also be **d**iscussed if appropriate. Once a management plan has been established, the members of the MET are encouraged to **e**xplain the cause of the call and subsequent management and **f**ollow-up plan to the medical and nursing staff, the patient and/or their next of kin. The subsequent frequency of monitoring of vital signs should also be discussed, as should the criteria for doctor re-notification. Finally, the members of the MET are encouraged to **g**raciously thank staff for their help with the MET call.

In addition to these guidelines, emphasis is placed on three principles regarding MET call management: first, always be helpful; second, never criticize the staff for making the call, or for the management of the patient; and third, always remain calm and concentrate on the management of the patient.

### Management of the 'hypoxic/tachypnoeic MET call'

Using the framework of the acronym '**A to G**', a plan was developed for the management of an episode of MET review initiated for a patient who is hypoxic or tachypnoeic (Table 5 in Additional file 1). Similar plans were developed for the management of the 'hypotensive MET call', the 'tachycardic MET call', the 'oliguric MET call' and, finally, the 'altered conscious state MET call'.

The aetiology and features of the common causes of the call are listed, as well as an approach to the management of each cause. In addition, criteria for seeking assistance or for notifying the intensivist are listed.

## Discussion

We conducted a study to determine the most common reasons for initiating 400 MET calls in a teaching hospital. In addition, we proposed minimum standards for the management of a MET call and developed a systematic framework for the assessment, management and referral of the various 'MET syndromes' that resulted in these calls.

Most of the literature related to METs has concentrated on their effects in reducing cardiac arrests and serious adverse events [1], primarily in single-centre studies.

Limited information exists on the cause of MET calls, and there is even less information on the process of assessment and management undertaken by the MET during an episode of MET review. To our knowledge, no information exists on a systematic approach to the management of such episodes.

Our analysis of 400 recent MET calls at the Austin Hospital revealed initial evidence supporting previous opinion that MET calls are likely to be made for a limited number of conditions [3] and that a MET syndrome or several MET syndromes exist [1]. Infections, pulmonary oedema, and arrhythmias featured as prominent causes of the 400 MET calls analysed. These syndromes have defined aetiologies and treatments.

At least two other studies have assessed the abnormalities leading to the activation of a MET service. In the original description of the MET, Lee and colleagues. [5] analyzed the cause of 522 MET calls, 148 of which were cardiac arrests and 62% of which occurred in the Emergency Department. The most common causes of MET calls in this study were acute respiratory failure, status epilepticus, coma, and severe drug overdose. Kenward and colleagues. [6] analysed 136 MET calls over a 12-month period and found that altered conscious state, hypoxia, tachypnea, hypotension and tachycardia were the commonest precipitants. An audit of 80 MET calls at the Northern Hospital in 2001 revealed that alteration in conscious state, hypotension, and noisy breathing were the commonest precipitants (Duke, G; unpublished data). These findings highlight the need to assess regional variations in the epidemiology of MET calls.

At least two other approaches exist that teach junior medical staff to manage acutely unwell hospital patients. The ALERT™ course was developed by staff affiliated with the University of Portsmouth [7,8]. The course provides an overall plan of assessment as well as approaches to the 'blue and breathless patient', 'the patient with a disordered conscious level' and 'the oliguric patient'. The '**A to G**' approach outlined in this article provides information about the aetiology, management and recommendations for referral and follow-up of patients with these and other syndromes.

The CCrISP course was developed by the Royal College of Surgeons (England) and is a two-day course aimed at surgical house officers [9]. The '**A to G**' approach outlined in this article is aimed primarily at medical and intensive care registrars and fellows, and incorporates acute deteriorations of both medical and surgical patients. It emphasizes the need to establish a diagnosis of the aetiology of the call and to establish a management and follow-up plan for the patient. In addition, we have included strategies to facilitate communication between members of the MET and the parent unit of the patient. Finally, we have emphasized the importance of not criticizing ward staff for initiating the call. Fear of criticism has been shown to be an obstacle for the activation of MET services [10,11].

Our study has several strengths and limitations. Our approach lends itself to education of the members of the MET and auditing of the MET review process. It is tailored for the team approach of the MET that involves an initial assessment and coordination of ongoing care. The other major strength of the approach is the ability to adapt it to the requirements of different hospitals. First, the 'MET syndromes' can be adapted according to the case mix and demographics of the patients at a given hospital to reflect the most common criteria and causes for the initiation of a MET call. Second, the details of the management plans can be altered according to local medical opinion and to reflect the level of experience of members of the MET. Third, it is possible to apply more objective and specific criteria for the notification of senior members of medical staff (for example, call the intensivist if the patient remains hypotensive despite receiving 3 litres of fluid).

The major limitation of the approach is that it has not been validated. We are currently implementing a detailed education programme at the Northern Hospital based on these recommendations that seeks to improve the documentation and outcome of patients who receive a MET review.

## Conclusion

We reviewed 400 MET calls and found that five syndromes accounted for essentially all MET calls observed; More than 90% of calls were associated with hypoxia, hypotension or altered conscious state. Sepsis, pulmonary oedema and arrhythmias were the most common triggers of MET calls and of the above syndromes. In response to these observations, we propose an approach that permits audit and standardization of the management of MET calls and provides an educational framework for the management of acutely unwell ward patients. Further evaluation and validation of the approach are required.

## Key messages

• 	Little information is currently available on what conditions trigger medical emergency team (MET) calls.

• 	We reviewed 400 MET calls and found that they could be reduced to five main syndromes.

• 	More than 90% of calls were associated with hypoxia, hypotension or altered conscious state.

• 	Sepsis, pulmonary oedema and arrhythmias were the most common underlying triggers for MET calls.

• 	Given the above observations, we propose minimum standards of response and a structured approach to managing MET calls.

## Abbreviations

ICU = intensive care unit; MET = medical emergency team.

## Competing interests

The authors declare that they have no competing interests.

## Authors' contributions

DJ, GD, JG, JB, AC and RB were responsible for the design of the study. DJ, AC and RB collected the data. DJ, GD and RB performed the data analysis. All authors performed the critical data review and prepared the manuscript. All authors read and approved the final manuscript.

## Supplementary Material

Additional File 1 A Microsoft Word file containing five tables: 'Calling criteria for Medical Emergency Teams' (Table 1); 'Common reasons for MET calls at the Austin Hospital' (Table 2); 'Proposed minimum criteria for managing a MET call' (Table 3); 'An approach to managing a MET call' (Table 4); 'Management of the 'hypoxic-tachypnoeic MET call" (Table 5). Click here for file
