# Supplementary material for: Medical Emergency Team syndromes and an approach to their management
Source: Crit Care. 2006 Feb 15;10(1):R30. doi: 10.1186/cc4821 (PMC1550805; doi:10.1186/cc4821)
Supplement: Additional File 1 — A Microsoft Word file containing five tables: 'Calling criteria for Medical Emergency Teams' (Table 1); 'Common reasons for MET calls at the Austin Hospital' (Table 2); 'Proposed minimum criteria for managing a MET call' (Table 3); 'An approach to managing a MET call' (Table 4); 'Management of the 'hypoxic-tachypnoeic MET call" (Table 5). [file cc4821-S1.doc]

Table 1: Calling Criteria for Medical Emergency Teams

- Staff member is worried about the patient
- Airway
  - Noisy breathing / stridor
- Breathing
  - Acute change in respiratory rate to < 8 or > 30 breaths / min
  - Acute change in pulse oximetry saturation to < 90% despite oxygen administration
- Circulation
  - Acute change in heart rate to < 40 or > 130 beats / min
  - Ischemic chest pain ¶
  - Acute change in systolic blood pressure to < 90 mmHg
  - Acute change in urinary output to < 50 mL in 4 hrs.
- Conscious state
  - Acute change in conscious state
  - Multiple seizures¶

¶ Indicates criteria specific for The Northern Hospital

**Table 2: Common reasons for MET calls at The Austin Hospital**

| **Cause of the MET call** | **Number of calls** |
| --- | --- |
| **Hypoxia / Increased respiratory rate**  Pulmonary oedema / fluid overload  Pneumonia / aspiration  Exacerbation chronic obstructive airways disease  Sepsis  Pulmonary embolism  Arrhythmia  Sputum plug, narcotized, acidemia, pleural effusion, tracheostomy blocked, atelectasis, intracranial event  No cause documented | **218**  66  52  16  11  11  12  30  20 |
| **Hypotension**  Sepsis  Bleeding / hypovolemia  Acute pulmonary oedema / myocardial ischemia  Arrhythmia  Cardiac arrest  Epidural related, Pulmonary embolism, anaphylaxis, vasovagal, Narcosis  No cause documented | **112**  30  28  15  10  4  13  15 |
| **Altered conscious state**  Sepsis  Stroke / Transient ischemic attach or Intracranial bleed  Seizure  Hypovolemia  Cardiogenic shock / acute coronary syndrome  Drug related  CO2 narcosis  Vasovagal, arrhythmia, cardiac arrest, encephalopathy, uremia, meningitis  No cause documented | **93**  13  13  11  8  6  5  5  21  12 |
| **Tachcyardia**  Arrhythmia  Sepsis  Acute pulmonary oedema / myocardial ischemia  Drug related  Hypovolemia  Respiratory distress  Pulmonary embolism, Epidural related, stroke  No cause documented | **77**  29  13  10  4  3  3  3  11 |
| **Oliguria**  Sepsis  Cardiogenic shock  Hypovolemia  Urinary tract obstruction  Drug related, hepatorenal syndrome, stroke  No cause documented | **31**  7  7  4  2  5  3 |

**Table 3: Proposed minimum criteria for managing a MET call**

- Determine the etiology of the deterioration
- Document the events surrounding the MET call

(A pre-formatted fluorescent yellow sticker is used at The Austin Hospital)

- Organize a management plan and appropriate medical follow-up
- Automatic medical referral for surgical patient subject to a MET call for a medical reason in cases where the patient remains on the ward¶
- Communicating with the parent unit (or their cover) that the MET has occurred
- Compulsory review of the patient by an Intensivist for a patient requiring two MET reviews in a seven day period ¶
- Communicating with the intensivist if the following criteria are fulfilled:
  - The patient remains unstable following initial resuscitation
  - The patient requires ICU or HDU admission
  - The patient may require ICU or HDU admission in the future
  - The patient has been admitted to ICU or HDU during this hospital admission
  - The members of the MET are unsure how to manage the patient (i.e. the members of the MET are worried about the patient).

¶ Criteria specific for Austin Hospital.

MET, Medical Emergency Team; ICU, Intensive Care Unit; HDU, High Dependency Unit.

**Table 4: An approach to managing a MET call**

**A**sk and **A**ssess

**A**sk the staff how you can help them
**A**sk about the reason for the MET call

**A**ssess for the etiology of the deterioration

**B**egin **b**asic investigations and resuscitation therapy

**C**all for help / **c**all consultant if needed

**D**iscuss, **D**ecide, and **D**ocument

**D**iscuss MET with parent unit / consultant

**D**iscuss advanced care planning if appropriated

**D**ecide where the patient needs to be managed

**D**ocument the MET and subsequent frequency of observations

**E**xplain: the cause of the MET, the investigations required and subsequent management plan

**F**ollow-up: which doctor to follow-up the patient? What are the criteria for doctor re-notification?

**G**raciously thank the staff at the MET

**Table 5: Management of the “Hypoxic – tachypneic MET call”**

**A**ssess for etiology

- Pulmonary edema / Cardiac failure (Past history of heart disease. Current evidence of myocardial ischemia, raised JVP, oedema, bilateral crepitations, cardiomegaly)
- Dependent atelectasis/collapse (Patient immobile, basal chest signs, recent surgery)
- Asthma / COAD (Wheeze, prolonged expiration, hyper-inflated chest)
- Sepsis anywhere. eg lung, kidney, wound, intra-abdominal.
- Pulmonary embolism – immobile, recent surgery, history of thrombo-embolism, tachycardia, ECG changes of right ventricular strain

**B**egin **b**asic investigations and resuscitation

- Administer oxygen and obtain portable CXR
- ECG, Cardiac enzymes, electrolytes
- Sepsis screen: FBE, CRP, blood, urine, sputum, wound.
- Consider ABG +/- lactate
- Pulmonary edema – **L**oop diuretic, **m**orphine, **n**itrates**, o**xygen**, p**osture,consider CPAP
- Dependent atelectasis/collapse – chest physiotherapy, humidified oxygen
- Asthma / COAD – bronchodilators, steroids, antibiotics ?BiPAP.
- Pulmonary embolism – V/Q scan or CTPA. Consider anti-coagulation.

**C**all for help

- SaO2 < 90% despite 10L inspired oxygen
- RR > 40, elevated PaCO2, altered conscious state

**D**iscuss & **D**ecide

- Is the patient stable or unstable?
- What is the management plan?
- Does the patient need ICU/HDU/surgery?
- Communicate with patient/Next of kin/parent unit/Intensivist
- What is the subsequent follow up plan?

**E**xplain

- Cause of the hypoxia and subsequent management plan.
- Subsequent observations required.

**F**ollow-up

- Who will follow-up the patient?

JVP = jugular venous pressure, COAD = chronic obstructive airways disease, WCC = white cell count, ECG = electrocardiogram, CXR = chest X-ray, ABG = arterial blood gas, FBE = full blood examination, CRP = C-reactive protein, V/Q = ventilation perfusion, CTPA = CT pulmonary angiogram, SaO2 = saturation oxygen, RR = respiratory rate, PaCO2­ = partial pressure of carbon dioxide, ICU = Intensive Care Unit, HDU = High dependency Unit.
